# Spatial and Temporal Variability in the Development and Potential Toxicity of *Phormidium* Biofilms in the Tarn River, France

**DOI:** 10.3390/toxins10100418

**Published:** 2018-10-17

**Authors:** Isidora Echenique-Subiabre, Maxime Tenon, Jean-François Humbert, Catherine Quiblier

**Affiliations:** 1 INRA, Sorbonne University, iEES Paris, 4 Place Jussieu, 75252 Paris CEDEX, France; isidora.echenique@gmail.com; 2 Unité Molécules de Communication et Adaptation des Microorganismes (MCAM, UMR 7245), Muséum National d’Histoire Naturelle, CNRS, Case 39, 57 rue Cuvier, 75005 Paris, France; maxime.tenon@gmail.com; 3 Department Sciences du Vivant, Paris Diderot University, 5 rue T. Mann, 75013 Paris, France

**Keywords:** river biofilms, benthic cyanobacteria, biofilm development, anatoxins, mass spectrometry, model II regression, coefficient of determination

## Abstract

Proliferation of *Phormidium* biofilms in rivers is becoming a worldwide sanitation problem for humans and animals, due to the ability of these bacteria to produce anatoxins. To better understand the environmental conditions that favor the development of *Phormidium* biofilms and the production of anatoxins, we monitored the formation of these biofilms and their toxins for two years in the Tarn River, biofilms from which are known to have caused the deaths of multiple dogs. As previously observed in New Zealand, *Phormidium* biofilm development occurred in riffle areas. The coverage of these biofilms at the bottom of the river exhibited strong spatial and temporal variations, but was positively correlated with water temperature and depth. Anatoxin-*a* was detected in less than 50% of the biofilms. The concentrations of these toxins in the biofilms exhibited high spatiotemporal variability, with the highest concentrations being recorded at the end of the summer period at the upstream sampling sites. These findings suggest that the maturity of the biofilms, combined with the local environmental conditions, have an impact on the production of anatoxin, making risk assessment for these benthic proliferations challenging.

## 1. Introduction

In recent years, the proliferation in rivers of biofilms dominated by filamentous cyanobacteria belonging to the genus *Phormidium* has been described as a potential risk for humans and animals, due to the production of neurotoxins by these microorganisms (reviewed by Quiblier et al. [1]). This proliferation of *Phormidium* biofilms is becoming widespread throughout river networks in New Zealand (NZ) [2,3], the United States (California) [4,5], and France [1,6]. It has been shown that *Phormidium* strains are able to synthesize mainly anatoxin-*a* (ATX) and homoanatoxin-*a* (HTX) [7,8,9,10], as well as the corresponding hydrogenated variants dihydroanatoxin-*a* (dhATX) and dihydrohomoanatoxin-*a* (dhHTX) [11]. ATXs are potent neuromuscular blocking agents that bind to neuronal nicotinic acetylcholine receptors, further inhibiting electrical transmission (reviewed by van Apeldoorn et al. [12]). Among the symptoms associated with ATX intoxication, the most prevalent are vomiting, epileptic seizures, muscular spasms in the limbs, paralysis of hind leg muscles, respiratory failure and, finally, death [7,8]. 

It is well established that ATXs are not secreted by cells and, consequently, that the unique method of intoxication is the ingestion of biofilms [1,10]. To date, the death of at least 40 dogs in France and >100 dogs in NZ has been attributed to neurotoxins produced by *Phormidium* since the beginning of the 2000s [8,13]. No evidence for the intoxication of humans or animals other than dogs can be found in the literature, which raises questions regarding this specificity for dogs. Among the hypotheses explaining this phenomenon, a particular affinity of dogs for biofilms dominated by *Phormidium* (hereafter referred to as *Phormidium* biofilms) has frequently been suggested, but has not been demonstrated. 

It has been shown, in NZ, that the potential toxicity of *Phormidium* biofilms depends, in part, on the importance of the development of these biofilms in rivers, which can be estimated by the percentage of biofilm coverage at the bottom of the river. Biofilm coverage is greatest during the summer period, when water temperatures are warm and the river flow relatively stable [10,14,15,16]. In addition to the variations in coverage, it has also been shown, in NZ, that the potential toxicity of biofilms exhibited great temporal and spatial variations because some *Phormidium* cells produce ATXs, while others do not [1,17]. 

In France, only two studies have been published on the toxicity of *Phormidium* biofilms in the Tarn and Loue rivers [8,18], and the environmental conditions favoring the development of these biofilms have not been studied. Moreover, no data are available on the spatiotemporal variations in the distribution and potential toxicity of these biofilms in the rivers; these data are needed to improve the monitoring of these cyanobacteria in rivers used for recreational activities, such as the Tarn River. To answer these questions, we monitored *Phormidium* biofilms in the Tarn River during the summer season in 2013 and 2014 with the following goals: (1) to identify the favorable biotopes and environmental conditions for the development of *Phormidium* biofilms in this river, and (2) to characterize the potential toxicity of these biofilms and the spatiotemporal variations in this potential toxicity. 

## 2. Results

### 2.1. Spatiotemporal Variations in the Coverage and Biomass of Biofilms in the Tarn River

**Figure 1:**
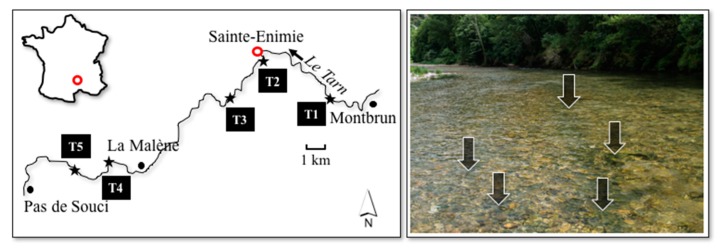
Locations of sampling sites (T1–T5) along the Tarn River, France. The image on the right shows a riffle area where *Phormidium* biofilms develop (black areas indicated by arrows).

A systematic visual examination of the river bottom in the whole sampling area (Figure 1) was performed during each sampling campaign; it was shown that the development of *Phormidium* biofilms occurs almost exclusively in the riffle areas and never in pools with low flow. The five riffle areas (T1–T5; Figure 1) selected for this study predominantly contained cobbles and boulders (5–26 cm length), and the main characteristics of these areas are described in Table 1. 

**Figure 2:**
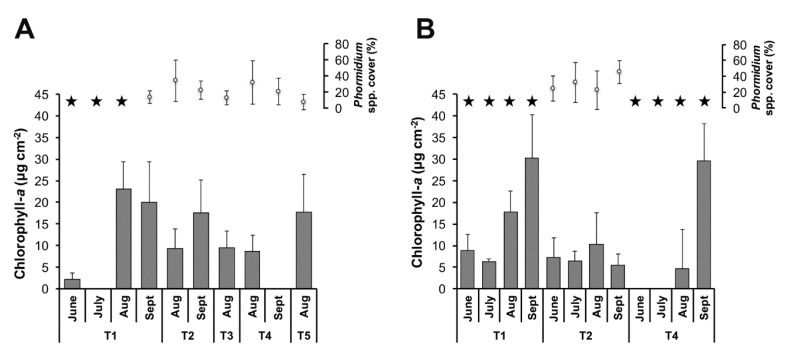
Variations in the monthly average values of the total chlorophyll-*a* concentrations (grey histograms) and in the coverage of the river bottom by *Phormidium* spp. (white circles) in the Tarn River in 2013 (**A**) and 2014 (**B**). In (**A**), sites T3 and T5 were sampled only in August 2013. The missing data points in the sampling months in (**A**), i.e., June and July in T2 and T4, and the biomass (Chl-*a*) values in July in T1 and September in T4, and in (**B**), i.e., June and July in T4, are due to (i) high flow velocities caused by high-rain events, which did not allow us to conduct sampling without putting the operators at risk, or/and (ii) the lack of visible *Phormidium* biofilms at the site during the sampling campaign. Stars represent percentage *Phormidium* spp. cover < 5%. Error bars are standard deviations.

In these riffle areas, significant spatial and temporal variations were found in the biofilm biomasses (expressed in µg of chlorophyll-*a* (Chl-*a*) per cm^2^) and in the *Phormidium* coverage during the two sampling years (ANOVA test, all *p* < 0.05) (Figure 2). In particular, the highest biomasses were recorded in August and September (Figure 2 and Table S1). When biofilms containing *Phormidium* were detected, microscopic analyses showed that cyanobacteria were highly dominant in these biofilms (>60% of the total biovolume) and that the genus *Phormidium* represents more than 97% of the total biovolume of cyanobacteria. Based on the sequencing of the 16S rRNA gene fragment using Illumina MiSeq, the dominant *Phormidium* operational taxonomic unit (with a 97% cut-off) of collected biofilms from the Tarn River displayed a 100% sequence similarity (Basic Local Alignment Search Tool, BLAST analysis on GenBank™, KF770970) with *Phormidium uncinatum* [19]. 

### 2.2. Relationship between Environmental Parameters and Phormidium Development in the Tarn River

**Figure 3:**
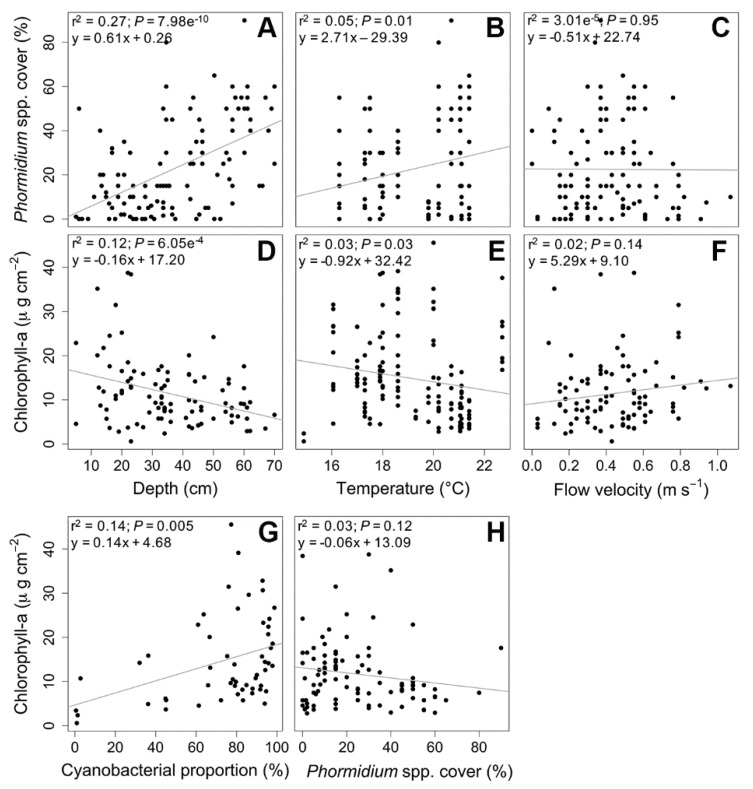
Relationship between *Phormidium* cover and depth (**A**), temperature (**B**), and flow velocity (**C**). Relationship between biofilm biomass (measured in terms of µg of chlorophyll-*a* per cm^2^) and depth (**D**), temperature (**E**), flow velocity (**F**), cyanobacterial proportion (**G**), and *Phormidium* cover (**H**). The corresponding linear regression equation and the coefficient of determination (*r*^2^) are shown in the top-left corner of each figure.

A positive correlation was found between depth and *Phormidium* coverage (*r*^2^ = 0.27, *p* = 7.98 × 10^−10^, Figure 3A) while, simultaneously, a weak but significant negative correlation was observed between depth and total biomass of the biofilms (expressed in µg Chl-*a* cm^−2^) (*r*^2^ = 0.12, *p* = 6.05 × 10^−4^, Figure 3D). As higher biomasses per surface unit indicate thicker biofilms, this finding suggested that the shallow areas were mostly characterized by thick cyanobacterial biofilms that covered the surfaces at the bottom of the river. When the depth increased, the thickness of the biofilms tended to decrease, while the percentage of coverage increased.

The positive correlation (*r*^2^ = 0.14, *p* = 0.005) observed between the biofilm biomass per surface unit and the proportion of *Phormidium* (Figure 3G) confirmed that the increase in the biofilm thickness was mainly due to cyanobacterial development. On the other hand, no significant correlation was observed between biofilm biomass and the coverage of *Phormidium* biofilms (Figure 3H).

Analysis of the variations in the flow rates during the two study periods showed that the summer of 2013 was characterized by very high flow rates at the beginning of the period (>40 m^3^ s^−1^ in May 2013), followed by a consistent decrease in the flow rate throughout the summer period (Figure S1). At the end of the summer (August–September), *Phormidium* coverage ranged between 5% and 40% at almost all sampling sites. In contrast, the summer of 2014 was characterized by several short episodes of high flow rates, and *Phormidium* coverage remained <5% at all sampling sites except site T2 (Figure 2 and Figure S1). 

### 2.3. Toxin Detection and Quantification

A total of 66 biofilm samples (three samples per site) were screened by PCR for the presence of selected gene fragments involved in the biosynthesis of ATXs, microcystins, and saxitoxins. No positive PCR signal was detected for the microcystin and saxitoxin gene fragments. On the other hand, the ks2 ATX gene fragment was detected in 15 of the 66 samples (Table 2). By LC-MS/MS analysis, 27 of the 63 samples analyzed tested positive for ATX (Table 2); HTX, dhATX, dhHTX, EpoxyATX, and EpoxyHTX were never detected. 

The overall congruence between the findings provided by the PCR and LC-MS/MS analyses was good (81%). All the positive results from the PCR analysis were confirmed by LC-MS/MS analysis, but some negative results from the PCR analysis were positive by LC-MS/MS analysis. Regarding the latter data, notably, the ATX concentrations were not quantifiable or very low in these samples. 

As shown in Table 2, ATX concentrations varied from 1.8 to 15.3 mg kg^−1^ FDW (freeze-dried weight) in biofilms, and all the biofilms that produced ATX were collected in August and September in 2013 and 2014. The highest ATX concentrations were always recorded in September in sites T1 and T2, in the upstream part of the river. 

**Figure 4:**
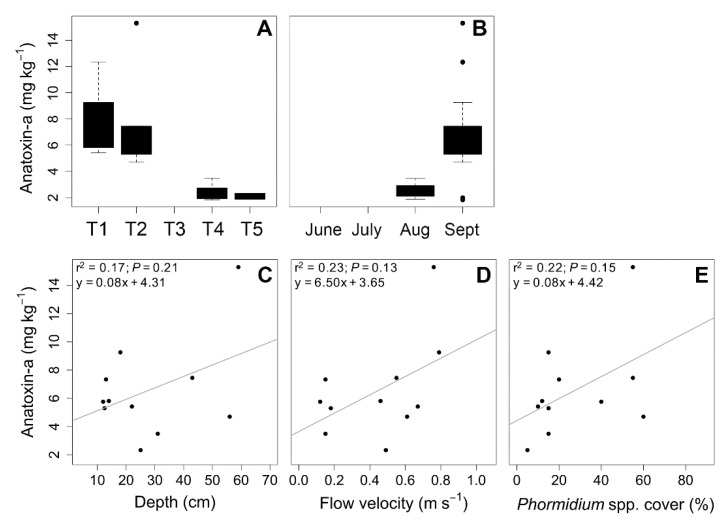
Variations in average anatoxin-*a* (ATX) concentrations by sampling site (**A**) and date (**B**) during the sampling campaigns performed in the Tarn River (2013–2014), and relationships between anatoxin-*a* concentration and depth (**C**), flow velocity (**D**), and *Phormidium* cover (**E**). The corresponding linear regression equation and coefficient of determination (*r*^2^) are shown in the top-left corner of each figure.

Finally, as summarized in Figure 4 from the data collected during the two-year period, (i) there was a clear decreasing gradient of ATX concentrations, from upstream to downstream (Figure 4A), and (ii) there was an increase in ATX concentrations at the end of the summer period (Figure 4B). Finally, when comparing ATX concentration and *Phormidium* coverage (Figure 4E), no significant correlation (*r*^2^ = 0.22, *p* = 0.15) was observed between the two parameters, nor with biomass (data not shown). Regarding water parameters, no significant correlation was observed between ATX concentration and depth or flow velocity (Figure 4C,D). 

## 3. Discussion

This study on the factors and processes promoting the development of river biofilms dominated by *Phormidium* cyanobacteria, and on the spatial and temporal variations in the production of ATXs in these biofilms, is the first of its kind to be performed outside NZ. 

Regarding the development of *Phormidium* biofilms, our data have shown that the environmental conditions that promote the development of these biofilms in the Tarn River are consistent with those previously described in NZ. For example, the highest biofilm coverage and biomass values were observed at flow velocities ranging between 0.3 to 0.8 m s^−1^ in the Tarn River, while Heath et al. [20] showed that the maximal coverage values in rivers in NZ were associated with flow velocities ranging between 0.6 and 1.1 m s^−1^. Similarly, the greatest development of *Phormidium* biofilms was observed when the water temperatures were ≥16 °C, while Heath et al. [15] showed that temperatures greater than 14 °C were correlated with increased *Phormidium* biofilm coverage in NZ. Finally, in both countries, *Phormidium* biofilms are mainly found in riffle areas on cobbles and boulders. The stability of these large substrates, in contrast to thin and more mobile substrates, may favor biofilm development when the flow velocity is high [20].

Interestingly, we found that depth (with measurements made between 0 and 70 cm) was positively correlated with biofilm coverage, but negatively correlated with the biofilm biomass (estimated per surface unit and thus expressing the thickness of the biofilm). We assume that light availability might explain this phenomenon; *Phormidium* biofilms that develop in shallow areas receive more light than those that develop at greater depths. At low depths, black biofilms are exposed to very large quantities of light, allowing the biofilms to grow in thickness. This thickness, in addition to the UV-protective molecules produced in these biofilms (i.e., scytonemin and mycosporine-like amino acids [21]), could protect the biofilms against UV radiation. For biofilms that develop at greater depths (35 to 70 cm), the formation of a thin layer over a large surface area is probably a more efficient method for light harvesting.

It is also very interesting to compare the trophic conditions in the Tarn River with those recorded in NZ rivers. As described in the Material and Methods section, the average concentrations of nitrate and total phosphorus (TP), during the study period in the Tarn River, were 1.46 mg L^−1^ and 0.02 mg L^−1^, respectively, while severe proliferation of *Phormidium* biofilms was observed in the rivers in NZ with average nitrate concentrations between 0.06 and 0.3 mg L^−1^, and an average phosphorus concentration of approximately 0.01 mg L^−1^. Moreover, high nutrient concentrations were recorded in Spain during benthic cyanobacterial development (i.e., 4 to 6 mg L^−1^ of nitrates and 0.4 mg L^−1^ of phosphates [22,23]). Loza et al. [24] observed and characterized different *Phormidium* morphogenotypes developing in several Spanish rivers with contrasting water qualities. All these data suggest that *Phormidium* biofilms can grow and proliferate in rivers exhibiting various trophic conditions, from oligotrophic to eutrophic. This trend is in contrast with that of the proliferation of planktonic cyanobacteria, which is clearly associated with eutrophic conditions in water bodies [25,26,27]. 

The low impact of trophic conditions on benthic cyanobacterial proliferations suggests that these events probably occur in a variety of rivers exhibiting physical and hydrological conditions suitable for the development of these biofilms. For example, during the summer of 2017, the proliferation of *Phormidium* biofilms was observed, for the first time, in several rivers in central France, including the Loire River (the longest French river), after a prolonged low-flow period (data not published). This is interesting to consider in the context of global climate change and effect of anthropogenic perturbations on aquatic ecosystems. Only a limited number of works were devoted to the study of toxic benthic cyanobacteria, despite the serious risk these microorganisms poses to animal and human health. We hypothesized that this may be explained by a recent and rapid increase of benthic proliferations. This is supported by the studies reporting toxic *Phormidium* proliferations over the last years in other geographic locations, including the Mundaú River in Pernambuco (Brazil) [28]; the Eel River [5], and other streams in California (USA) [4].

The potential toxicity of *Phormidium* biofilms in the Tarn River was assessed first by a PCR-based method, then by LC-MS/MS, to detect ATX. Only the ks2 gene fragment, involved in the biosynthesis of ATX, was detected by PCR, while negative signals were observed for gene fragments involved in microcystin and saxitoxin biosynthesis. These latter gene fragments (associated with microcystin and saxitoxin synthesis) were screened because other cyanobacterial species (e.g., *Dolichospermum*), that are known to produce these toxins, can be found in association with *Phormidium* in biofilms, and because microcystins [5,29,30,31] and saxitoxins [29] have been previously detected in *Phormidium* extracts.

With regard to ATX, all the positive results obtained by PCR were confirmed by mass spectrometry, but false negative results were also obtained by PCR. Previous studies have shown that the existence of polymorphisms in the ATX gene cluster [18,32] can reduce the efficiency of PCR primers, leading to false negative results. Similarly, partial inhibition of the PCR might also lead to false negative results when the targeted genes are present at low quantities, which was probably the case in our study, in which the false negative results in the PCR were associated with very low amounts of ATX being detected by LC-MS/MS.

Unlike the results from NZ, where HTX and di-hydro derivatives were the dominant toxins in *Phormidium* biofilms [17], but consistent with data obtained by Bouma-Gregson et al. [5] in benthic biofilms from the Eel River in California, only ATX was detected in the Tarn River samples. The ATX concentrations in the biofilms from the Tarn River were lower than those estimated for strains isolated from the Loue River in France [8], but were in the same concentration range as that estimated in rivers in NZ [17]. As shown in the Material and Methods section, it is likely that the ATX concentrations were underestimated in the Tarn River biofilms, due to partial extraction of this ATX.

Our results revealed no correlation between ATX concentration and *Phormidium* coverage nor biomass, which was consistent with the results previously obtained in NZ rivers [15]. A recent study performed by Wood and collaborators [33] showed that *Phormidium* cover and toxin concentration were nearly linear at coverages above 10%. In order to better understand this relationship, further studies should be conducted. This lack of correlation between biofilm biomass and ATX concentration is interesting to consider in the context of the implementation of monitoring programs for these benthic cyanobacteria, by showing that biofilm biomass cannot be used as a proxy for ATX concentration. 

Interestingly, our data have revealed the existence of (i) an upstream–downstream negative gradient in these ATX concentrations in the Tarn River, and (ii) an increase in ATX concentrations during the summer season (from June to September). While significant temporal shifts occurring on a weekly basis were observed in rivers in NZ (i.e., from 27-fold to 80-fold variability) (see review by Wood and Puddick [34]), such an increase in ATX concentrations during the summer season has not been previously described. This finding suggests that ATX production occurs mainly in mature biofilms in the Tarn River. Consistent with this hypothesis, two high-flow events were recorded in June 2013 and July 2014, probably resulting in the development of new biofilms, as previously shown in numerous rivers [1,3,35,36]. Moreover, the dominance of diatoms in the biofilms collected immediately after these high-flow events also suggested the development of new biofilms. Given that no high flow event was recorded until the final sampling campaign in September, it can be postulated that the biofilms sampled in late August and in September were mature biofilms. We have no data that explain the upstream–downstream decreasing gradient in ATX concentrations in September. However, it has been shown by Wood and Puddick [34] that *Phormidium* populations contain a mix of ATX-producing and non-ATX-producing filaments; therefore, it is possible that variations in environmental conditions from upstream to downstream may lead to differential selection of these two types of filaments in these areas. 

## 4. Material and Methods

### 4.1. Sites and Sample Collection

The Tarn River is located in a highly touristic area in the south of France; this river is one of the most famous sites in France for canoeing during the summer. The Tarn River has a pluvionival hydrological regime, and is a tributary of the Garonne River. The water of the river is classified as having globally good quality, and as oligotrophic [37]. Sampling in the river was performed between June and September, in 2013 and 2014, in a section of ~25 km where dog mortalities linked to the consumption of cyanobacterial biofilms had been observed [18]. After visual examination of the bottom of the river section, sampling sites were chosen in five riffle areas where *Phormidium* biofilms were detected (T1 to T5; Figure 1). These sites were characterized by shallow waters (generally <60 cm), flow velocities > 0.3 m s^−1^, and a substrate dominated by cobbles and boulders (5 to 26 cm in length) (Table 1). Sites T3 and T5 were sampled only in August 2013 as part of an expanded monitoring campaign.

On each site (except for site T1 in June 2013) a grid of 10 m × 10 m was set up from the shoreline and, within this grid, ten sampling points were randomly selected [17]. At each point, a single cobble with a *Phormidium* biofilm was sampled using an underwater viewer. Biofilms were removed from the cobble using sterile tweezers, and subsamples were obtained for DNA and toxin analyses. In addition, a known area of the biofilm (7.07 cm^2^) was sampled for chlorophyll-*a* quantification. All these samples were placed in a 15 mL Falcon tube. Finally, 1 cm^2^ of the biofilm was also fixed with Lugol’s iodine solution for subsequent microscopic identification and enumeration. All the samples were stored chilled in the dark and subsequently frozen (−20 °C), except for the samples used for microscope analysis, which were stored at 4 °C. 

For site T1 in June 2013, as the coverage of the substrate by *Phormidium* biofilms was very low (<5% of the river substrate), three sampling points, located on a transect parallel to the water’s edge and positioned 1.5 m from the shoreline, were sampled. At each point, all cobbles (5 to 20 cm length) visible in a single field of view of the underwater viewer (707 cm^2^) were collected. The cobbles were scrubbed, and the biomass was collected in 150 mL of river water. Aliquots (5 mL) were filtered for Chl-*a* (GF/C Whatman) and DNA extraction (Polycarbonate, 0.2 μm GTTP; Millipore, Bedford, MA, USA). Subsamples (1 mL) were fixed with Lugol’s iodine solution. The remainder of the procedure was the same as that used for the other samples.

Rivers are very dynamic systems and, thus, biofilms that develop in these ecosystems are subjected to changes in water flow/velocities. The missing data points in Figure 2 are a consequence of (i) high flow velocities caused by high-rain events, which did not allow us to conduct sampling without placing the operators at risk; or/and (ii) the lack of *Phormidium* biofilms at the site during the sampling campaign.

### 4.2. Estimation of the Extent of Phormidium Proliferation

The extent of the development of *Phormidium* biofilms at the five different sites was determined by estimating the percentage of substrate covered by *Phormidium* biofilms (biofilm coverage) and the biomass of the biofilms per surface unit, using chlorophyll-*a* as a proxy. 

*Phormidium* biofilm coverage was estimated at ten randomly selected points within the grid. At each point, the coverage was visually estimated through an underwater viewer independently by two operators. In this paper, the *Phormidium* biofilm coverage of a site represents the average value of the ten sampling points.

Chl-*a* was extracted from the biomass samples or glass fiber filters (from biofilms collected at site T1 in June 2013) with methanol (10 mL) in Falcon tubes (15 mL) covered with aluminum foil, following a previously described protocol [6]. Biofilm biomass was estimated and expressed in µg of Chl-*a* per cm^2^, according to a protocol described previously [6]. 

### 4.3. Environmental Parameters

At each sampling site water temperature and pH were measured using a multi-parameter probe (model HI 98130, HANNA Instruments, Woonsocket, RI, USA). Then, at each sampling point, flow velocity and depth were measured with a Flow Probe FP111 (Global Water, College Station, TX, USA). In addition, the flow rate (Figure S1) was obtained from two survey stations (Bédouès and Mostuéjouls) located 28 km upstream and 38 km downstream, respectively, from Sainte-Enimie (T2, Figure 1) (http://hydro.eaufrance.fr). The average concentrations of nitrates and TP measured at Quézac (~6 km upstream from Montbrun) were 1.46 mg L^−1^ (NO_3_-N) and 0.017 mg L^−1^, respectively, during the sampling period (data extracted from the French database SIE Adour-Garonne; Système d’Information sur l’Eau Adour-Garonne; http://adour-garonne.eaufrance.fr/accesData/). 

### 4.4. Study of the Composition of Photosynthetic Communities Present in the Biofilms

Samples preserved with Lugol’s iodine were homogenized briefly (Ultra-Turrax T25, IKA, Staufen, Germany; 3 × 2 s, 9.5 min^−1^) to break up the filaments but avoid cell damage. The samples were diluted in Milli-Q water, and identification and enumeration were performed using a light microscope (200× magnification, Nikon Optiphot-2, Tokyo, Japan) and a Malassez chamber (Marienfeld, Lauda-Königshofen, Germany). Counting, identification, and biovolume estimation of the samples were performed as previously described [6].

### 4.5. DNA Extraction and Estimation of Toxic Potential

Three biofilm samples from each sampling site were lyophilized (FreeZone2.5, Labconco, Kansas City, MO, USA). Lyophilization was performed directly from frozen biofilm material (−20 °C) contained in Falcon tubes (15 mL). Falcon tubes were kept protected from light and biofilm samples were freeze-dried (≤−50 °C and 0.13 mBar vacuum pressure) for 24 to 48 h, depending on biomass and water content. DNA was extracted from ca. 50 mg of lyophilized biofilm material using a Power Biofilm^®^ DNA Isolation Kit (MOBIO, Carlsbad, CA, USA) following the manufacturer’s instructions. One ATX-producing strain, *Phormidium favosum*, isolated from the Loue River (PMC 240.04) was also analyzed as a control strain. Nucleic acid concentrations were determined by spectrophotometry (Nanodrop 1000, Thermo Fisher Inc., Wilmington, DE, USA), and the samples were stored at −20 °C. The ATX production potential of the environmental samples was assessed by PCR-based targeting of the ks2 DNA fragment (~400 bp region; [38]). In addition, we tested the potential toxicity of the biofilms by screening for genes involved in the biosynthesis of microcystin (mcyA, [39]) and saxitoxin (stxG, [40]), and for each PCR, a toxic strain was used as a positive control (*Microcystis aeruginosa* PMC 00.11 and *Aphanizomenon gracile* PMC 638.10, respectively). 

### 4.6. Extraction of Anatoxins

Triplicates of the lyophilized biofilm material (100 mg) were resuspended in 10 mL of distilled water (Milli-Q) containing 0.1% formic acid. Strain isolates (~15 mg) were resuspended in ~1.5 mL of distilled water (Milli-Q) containing 0.1% formic acid. Samples were extracted according to a previously described protocol [41] with some modifications: the samples were directly sonicated (VibraCell, Sonics, Newtown, CT, USA) twice for 3 min at 80% amplitude in glass assay tubes, and then transferred to 15 mL Falcon tubes for centrifugation (3220× *g*, 30 min at 4 °C). The supernatants were concentrated by lyophilization and rehydrated with water, and the pH was adjusted to 9.0 with aqueous NH_4_OH. Solid-phase extraction was performed using a C18-SD column (3M^TM^ Empore^TM^ high-performance extraction disk cartridges, 7 mm/3 mL; Sigma-Aldrich, St. Quentin Fallavier, France), previously washed with 250 μL of MeOH and then with 500 μL of H_2_O. Each sample was loaded by gravity, and the column was washed successively with 500 μL of H_2_O and 500 μL of 10% MeOH/H_2_O (*v*/*v*). The toxins were eluted with 300 μL of MeOH. After solvent evaporation under reduced pressure (CentriVap, Labconco, Kansas City, MO, USA), the residues were dissolved in 50 μL of 0.1% formic acid/H_2_O (*v*/*v*). To calculate the percentage of ATX loss during the extraction procedure, two replicate samples were enriched with a known ATX concentration, and these samples were subjected to all the steps of extraction until quantification. The ATX recovery percentage was 35% ± 3.5 (average of two enriched samples), implying that the ATX concentration was probably underestimated in our study.

### 4.7. Detection of Anatoxins

The LC-MS/MS analyses were performed by a combination of HPLC and mass spectrometry. The HPLC system was an Ultimate 3000 from Dionex (Thermo Fisher, Waltham, MA, USA) coupled to a QTRAP LC/MS/MS system from ABI Sciex (Courtaboeuf, France), which integrates a hybrid quadrupole-linear ion trap mass spectrometer with an electrospray ionization (ESI) source. The analytical LC column used for cyanotoxin separation was a reversed-phase C12 Jupiter 4U Proteo 90A 4 micron (250 mm × 1.0 mm i.d.) from Phenomenex (Le Pecq, France). The temperature was set at 20 °C. The composition of the mobile phase was as follows: water (A) and acetonitrile (B), both containing 0.1% of formic acid. Chromatographic separation was performed by gradient elution (50 min): isocratic for 15 min at 100% A, followed by a gradient to 60% B over 10 min and, finally, at 100% A. The mobile phase flow rate was 70 µL min^−1^, and the injection volume was 10 µL. Collision-induced dissociation (CID) in the ion trap MS was performed. The ESI source of QTRAP was operated with the following optimized values of source-dependent parameters: ion spray voltage (IS), 5500 V; curtain gas (CUR), 20; collision gas (CAD), high; declustering potential (DP), 20; entrance potential (EP), 10; collision energy (CE), 20; and collision cell potential (CXP), 15. The mass spectrometer was operated in multiple reaction monitoring (MRM, 200.0 ms) mode, detecting in positive mode, to analyze the following transitions: ATX (*m*/*z* 166 > 149, *m*/*z* 166 > 105), HTX (*m*/*z* 180 > 163, *m*/*z* 180 > 145), dhATX (*m*/*z* 168.0 > 56), dhHTX (*m*/*z* 182 > 57), EpoxyATX (*m*/*z* 182 > 98) and EpoxyHTX (*m*/*z* 196 > 140). The instrument was calibrated with dilutions of authentic standards of ATX and HTX in 0.1% formic acid. 

### 4.8. Statistical Analysis

Statistical analyses were performed using the statistical software package R 3.1.0 (R Development Core Team, Vienna, Austria [42]). Normality was checked using the Shapiro–Wilk test, and homoscedasticity was checked with the Breusch–Pagan test (package “lmtest”; [43]). The effects of sampling site and date on biomass (Chl-*a*, cyanobacterial biovolume proportions and percentage *Phormidium* spp. coverage) were analyzed using two-way ANOVA, followed by multiple comparisons using Tukey’s honestly significant difference (HSD) post hoc test. 

The relationship analysis by type II linear regression (package “lmodel2”; [44]) was performed using the major axis method. When no normalization was possible, relationships were analyzed by type II linear regression using ordinary least squares. The coefficients of determination (*r*^2^ values) presented throughout the manuscript correspond to squared correlation coefficients from type II regression analysis. Differences were considered significant when *p* < 0.05. 

## Figures and Tables

**Table 1:** Average values of the physicochemical and biological parameters measured during the study period at the five sampling sites in the Tarn River.

Year	Site	Flow Velocity (m s^−1^)	Depth (cm)	pH	T (°C)	Chlorophyll-*a* (μg cm^−2^)	*Phormidium* Cover (%)
2013	T1	0.6 ± 0.3	21.5 ± 4.8	7.9 ± 0.1	19.6 ± 2.9	19 ± 10	13.9 ± 7.8
	T2	0.3 ± 0.2	27.5 ± 16.2	7.8 ± 0.1	19.7 ± 1.1	13.4 ± 7.4	28.2 ± 20.4
	T3	0.5 ± 0.2	27.9 ± 11.9	8.1 ± 0	21.1 ± 0	9.4 ± 4	12.9 ± 8.5
	T4	0.3 ± 0.1	44.4 ± 17.4	8 ± 0.1	18.3 ± 2	8.6 ± 3.8	26.3 ± 22.5
	T5	0.6 ± 0.2	35.5 ± 9.9	8.1 ± 0	17.9 ± 0	17.7 ± 8.7	7.5 ± 9.8
2014	T1	0.6 ± 0.2	20.3 ± 13	8.4 ± 0.3	18.6 ± 1.2	16.4 ± 10.4	2.5 ± 3.2
	T2	0.4 ± 0.2	46.9 ± 15	8.4 ± 0.2	19.3 ± 1.9	7.9 ± 4.5	31.2 ± 21.8
	T4	ND *	ND	8.1 ± 0.3	18.2 ± 2.1	24.8 ± 9.8	<5

ND *: Not determined.

**Table 2:** Anatoxin detection in the *Phormidium* biofilms from the Tarn River performed by PCR-based targeting of the ks2 fragment responsible for the biosynthesis of the ATX gene and by liquid chromatography-mass spectrometry. Three samples were tested for each site with the following criteria: (i) the same samples were used for PCR and LC-MS/MS when the biofilm biomasses were sufficient to perform the two analyses, and (ii) different samples were used for PCR and LC-MS/MS analysis of biofilms when the biomasses were low (indicated by * in the table). All toxin measurements are given as mg kg^−1^ of lyophilized weight. nq = positive but non-quantifiable. nt = not tested.

	Site	Month	Sample	PCR Detection	LC-MS/MS Detection	LC-MS/MS Quantification (mg kg^−1^)
**Tarn 2013**	**T1**	June	S1	−	nt	
			S2	−	nt	
			S3	−	nt	
		July	S1 *	−	−	
			S2 *	−	−	
			S3 *	−	−	
		August	S1 *	−	−	
			S2	−	−	
			S3	−	−	
		September	S1 *	+	+	5.42
			S2	+	+	9.26
			S3	+	+	5.81
	**T2**	August	S1 *	+	+	nq
			S2 *	+	+	nq
			S3 *	−	+	nq
		September	S1 *	+	+	7.34
			S2	+	+	5.76
			S3	+	+	5.30
	**T3**	August	S1 *	−	−	
			S2 *	−	−	
			S3 *	−	−	
	**T4**	August	S1 *	−	−	
			S2 *	−	+	3.49
			S3 *	−	+	nq
		September	S1	−	−	
			S2 *	−	+	nq
			S3	−	+	nq
	**T5**	August	S1 *	−	+	2.33
			S2	−	−	
			S3	−	+	1.87
**Tarn 2014**	**T1**	June	S1	−	−	
			S2	−	−	
			S3	−	−	
		July	S1	−	−	
			S2	−	−	
			S3	−	−	
		August	S1	−	−	
			S2	−	−	
			S3	−	−	
		September	S1	+	+	12.33
			S2	+	+	nq
			S3	−	+	5.93
	**T2**	June	S1	−	−	
			S2	−	−	
			S3	−	−	
		July	S1	−	−	
			S2	−	−	
			S3	−	−	
		August	S1	−	+	nq
			S2	+	+	nq
			S3	+	+	nq
		September	S1	+	+	4.70
			S2	+	+	15.30
			S3	+	+	7.45
	**T4**	June	S1 *	−	−	
			S2	−	−	
			S3	−	−	
		July	S1	−	−	
			S2	−	−	
			S3	−	−	
		August	S1	−	−	
			S2	−	+	nq
			S3	−	−	
		September	S1	−	+	1.84
			S2	−	+	1.99
			S3	−	−	

+ detected; − non detected.

## Supplementary Materials

The following are available online at http://www.mdpi.com/2072-6651/10/10/418/s1, Figure S1: Flow rates of Tarn River in 2013 (A) and 2014 (B) measured at Bédouès (dotted line) located 28 km upstream and Mostuéjouls (solid line) located 38 km downstream from Sainte-Enimie, respectively. Arrows show sampling days. Table S1: Statistical analyses of cyanobacterial proportion, chlorophyll-*a* and percentage *Phormidium* cover of the Tarn River in 2013 and 2014. Asterisk denotes interaction between factors.
