# Supplementary material for: Spatial and Temporal Variability in the Development and Potential Toxicity of Phormidium Biofilms in the Tarn River, France
Source: Toxins (Basel). 2018 Oct 17;10(10):418. doi: 10.3390/toxins10100418 (PMC6215143; doi:10.3390/toxins10100418)
Supplement: Supplementary file 1 [file toxins-10-00418-s001.zip › toxins-357114-supplementary.docx]

Supplementary Materials: Spatial and Temporal Variability in the Development and Potential Toxicity of Phormidium Biofilms in the Tarn River, France

Isidora Echenique-Subiabre, Maxime Tenon, Jean-François Humbert
and Catherine Quiblier

**Table S1.** Statistical analyses of cyanobacterial proportion, chlorophyll-*a* and percentage *Phormidium* cover of the Tarn River in 2013 and 2014. Asterisk denotes interaction between factors.

|  | **Tarn 2013** | | **Tarn 2014** | |
| --- | --- | --- | --- | --- |
| **Variable** | **Two-Way ANOVA Test (*P*)** | **Tukey Test (*P*)** | **Two-Way ANOVA Test (*P*)** | **Tukey Test (*P*)** |
| **Cyanobacterial Proportion (%)** | Site (0.009)  Month (<0.001)  Site*Month (0.72) | T1-T5 (0.014)  T2-T5 (0.018)  June-July (<0.001)  June-Aug (<0.001)  June-Sept (<0.001) | Site (0.93)  Month (0.04)  Site*Month (0.17) | July-Aug (0.035) |
|  |  | July-Sept (0.01)  Aug-Sept (0.004) |  |  |
| **Chlorophyll-*a* (µg cm^-2^)** | Site (<0.001)  Month (<0.001)  Site*Month (0.008) | T1-T3 (0.003)  T1-T4 (<0.001)  T3-T5 (0.048)  T4-T5 (0.02)  June-Aug (<0.001)  June-Sept (<0.001) | Site (<0.001)  Month (<0.001)  Site*Month (<0.001) | T1-T2 (<0.001)  T1-T4 (<0.001)  T2-T4 (<0.001)  June-Sept (0.049)  July-Sept (<0.001)  Aout-Sept (0.038) |
| ***Phormidium* Cover (%)** | Site (0.007)  Month (0.036)  Site*Month (0.95) | T2-T5 (0.02)  T4-T5 (0.04)  Aug-Sept (0.036) | Site (<0.001)  Month (0.03) | T1-T2 (<0.001)  Aug-Sept (0.038) |

**
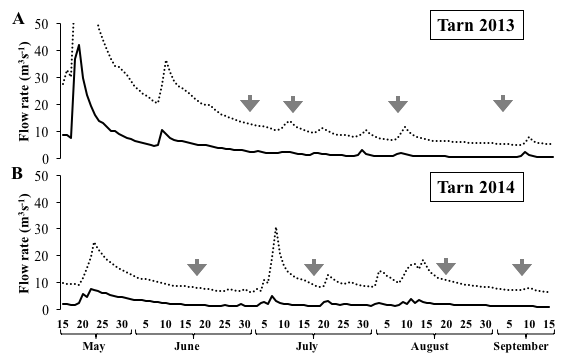
**

**Figure S1.** Flow rates of the Tarn River in 2013 (A) and 2014 (B) measured at Bédouès (doted line) located 28 km upstream and Mostuéjouls (solid line) located 38 km downstream from Sainte-Enimie respectively. Arrows show sampling days.
